# Morphological analysis when morphemes are hard to detect: Evidence from infixation and reduplication

**DOI:** 10.3758/s13423-026-03026-y

**Published:** 2026-10-23

**Authors:** Dave Kenneth Tayao Cayado, Kathleen Rastle

**Affiliations:** Department of Psychology, Royal Holloway, University of London, Egham, UK

**Keywords:** Morphology, Morphological processing, Visual word recognition, Reading, Tagalog

## Abstract

There is substantial psycholinguistic evidence that skilled reading involves morphological analysis, whereby morphologically complex words like *teacher* are recognised via constituent morphemes {teach}+{-er}. The ability to recognise morphemes is vital as it allows readers to interpret both familiar and unfamiliar words. However, over 50% of the evidence for this claim comes from studies of suffixation in English, where morphemes typically have a relatively consistent surface form across exemplars (*clean* in *cleaner, cleanliness*; *-ed* in *walked*, *jumped*). It remains unclear whether morphological analysis is also robust when morphemes lack a consistent surface form. Using the morpheme interference paradigm in Tagalog, we test whether infixes and reduplicants are detected during prelexical processing. Infixes are inserted within a stem and therefore reduce the consistency of its surface realisation (e.g., *tawag* → *t-in-awag* ‘called’), while reduplicants involve partial or full copying of a stem and therefore lack a consistent surface realisation (e.g., *tawag* and *lundag* become ***ta****tawag* ‘will call’ and ***lu****lundag* ‘will jump’, where *ta-* and *lu-* are instances of the same reduplicant morpheme). We found robust morpheme interference effects in both accuracy and RTs. Specifically, nonwords with morphological structure induced significantly lower accuracy and longer RTs than nonwords without. Our findings show skilled readers’ acquired sensitivity to these sophisticated morphological structures and motivate refinements to theories of morpheme learning.

**Supplementary Information:**

The online version contains supplementary material available at https://doi.org/10.3758/s13423-026-03026-y.

## Introduction

In the majority of the world’s languages, words are not atomic: they are composed of smaller meaning-bearing units called *morphemes* that combine and recombine to create new words. For example, the English word *mistrustfulness* comprises four morphemes—*mis-*, -*trust-*, -*ful-*, and *-ness*—each contributing a distinct component of meaning. Crucially, most unfamiliar words that readers encounter are combinations of morphemes, suggesting that morphological knowledge plays a critical role in vocabulary growth (e.g., Brysbaert et al., 2016; Korochkina et al., 2024; Rastle & Taylor, 2018). How, then, do readers build a representation of morphemes? The dominant account is grounded in statistical learning theory: Readers extract orthographic sequences that reliably contribute to the meanings of words through their text experience (e.g., Korochkina & Rastle, 2025; Lelonkiewicz et al., 2020; Plaut & Gonnerman, 2000; Tamminen et al., 2015). For instance, the English suffix -*ed* in *walked*, *jumped*, and *kicked* consistently marks past tense, allowing readers to identify this recurring orthographic chunk as a meaningful unit.

This acquired morphological knowledge is deployed rapidly during skilled reading. Readers routinely analyse morphologically complex words in terms of their constituent morphemes during visual word recognition (see Rastle & Davis, 2008, for a review). Words are recognised more quickly when they contain high-frequency stems (e.g., *unreal* vs. *refuel*; Cole et al., 1989; Taft & Ardasinski, 2006). Morphologically related primes facilitate recognition of target words (e.g., darkness–DARK) relative to unrelated primes (e.g., fullness–DARK; see Rastle & Davis, 2008). These findings provide evidence that morpheme representations are accessed during routine lexical processing.

Morphological analysis has also been investigated using the morpheme interference paradigm, where participants judge the lexicality of morphologically structured nonwords and orthographic controls. In this paradigm, morphologically structured nonwords are formed by combining an existing stem and an existing affix that do not typically go together (e.g., *gasful*), while orthographic controls are created by replacing one letter in the affix (e.g., *gasfil*; Crepaldi et al., 2010). Existing studies have shown that readers reject morphologically structured nonwords such as *gasful* more slowly and less accurately than nonwords lacking such morphological structure (e.g., *gasfil*; Caramazza et al., 1988; Crepaldi et al., 2010; Korochkina et al., 2026). The same morpheme interference effects have also been observed in Spanish (Carden et al., 2019), Italian (Caramazza et al., 1988), French (Casalis et al., 2015), Swedish (Jarvella & Wennstedt, 1993), and Hebrew (Yablonski & Ben-Shachar, 2016). These findings provide evidence that morpheme analysis is not restricted to lexical processing but also arises during the processing of morphologically structured nonwords.

One limitation of this research literature is that it is based on a very narrow set of conditions: for example, a recent review shows that over 50% of the evidence pertaining to morphological processing in visual word recognition comes from suffixation in English and closely related European languages (Cayado & Rastle, 2025). Suffixes in English and similar European languages are typically realised as consistent orthographic forms (e.g., *-ed* in *walked*, *jumped*). Further, they typically attach to full stems or to stems with minor orthographic alterations (e.g., missing ‘e’, as in *adorable*; McCormick et al., 2008). This raises a critical question: does morphological analysis remain robust when surface orthographic forms are inconsistent?

We address this question by examining two such cases in Tagalog: infixation and reduplication, which have important grammatical functions in the language. Tagalog has two inflectional infixes, namely *<in>*, which marks perfective aspect, and *<um>*, which marks agent voice. Infixes disrupt the consistent realisation of a stem because they are inserted *within* the stem, specifically after the first consonant. For example, the stem *tawag* becomes *t-in-awag* ‘called’ and *t-um-awag* ‘to call’ when infixes *-in-* and *-um*- are inserted to mark completed actions and agent of the actions, respectively. Reduplication involves partial or full copying of a stem to mark various inflectional and derivational functions, and therefore, the same morpheme is realised in dozens of ways. For example, the stems *tawag* and *lundag* become ***ta****tawag* ‘will call’ and ***lu****lundag* ‘will jump’ in partial reduplication, where *ta-* and *lu-* are instances of the same reduplicant morpheme marking ‘incomplete action’. Full reduplication can transform nouns to adverbs (e.g., *gabi* ‘night’ and *araw* ‘day’ become ***gabi****gabi* ‘every night’ and ***araw****araw* ‘every day’) or nouns to adjectives (e.g., *bahid* ‘stains’ becomes *bahid bahid* ‘full of stains’). In the present study, we investigate whether these complex words are subject to morphological analysis during prelexical processing.

Existing theoretical accounts have conceptualised morphology as a statistical learning problem, such that skilled readers discover orthographic sequences that contribute predictably to the meanings of words through their text experience (Korochkina & Rastle, 2025; Plaut & Gonnerman, 2000; Rastle et al., 2000). Stems occur repeatedly in words that have similar meanings (e.g., *cleanly*, *cleanliness*, *cleaner*) and affixes alter the meanings of words in predictable ways (e.g., *teacher*, *builder*, *publisher*)—and these forms of systematicity have consequences for learning. Studies using artificial laboratory learning paradigms show that readers develop representations of affix-like orthographic chunks that occur frequently at the beginning or end of novel words (Lelonkiewicz et al., 2020). Further, the robustness of these representations increases as a function of the number of novel words in which these chunks occur (type frequency) and the consistency in which they modify the meanings of those words (Tamminen et al., 2015). Evidence regarding the morpheme knowledge of skilled readers paints a similar picture. One recent study found that skilled readers’ knowledge of English prefixes and suffixes as measured in online lexical processing is positively associated with the type frequency of these morphemes and their semantic consistency (Korochkina et al., 2026).

However, the claim that morphological knowledge is acquired by tracking how the same orthographic forms reoccur in words with similar meanings may not extend to other morphological types. First, in *infixation*, the typical surface realisation of the stem is disrupted. For instance, the stem *tawag* ‘call’ has the same orthographic form across exemplars in prefixation (e.g., *nag-tawag* ‘called (someone)’; *pag-tawag* ‘way of calling’) and suffixation (e.g., *tawag-in* ‘to call’; *tawag-an* ‘call for’), but the stem is obscured when an infix is inserted (e.g., *t-in-awag* ‘called’; *t-um-awag* ‘to call [someone]’). The disruption to stems that arises because of infixation means that it may become difficult to detect orthographic forms that reoccur in words with similar meanings. Readers need to learn that *tawag* and *t–awag* are different realisations of the same stem. Second, in *reduplication*, the surface realisation of the morpheme is inconsistent. For instance, the reduplicants *ta-*, *ti-*, and *lu-* in partially reduplicated words *ta-tawag *‘will call’, *ti-tili *‘will scream’, and *lu-lundag *‘will jump’, respectively, are exactly the same morpheme carrying the same grammatical function. These reduplication cases raise an important question about whether readers can build representations of morphemes when there is no stable orthographic form that maps onto meaning. To learn this morpheme, readers would need to track the *process* of copying the CV cluster or the entire stem, rather than the surface form itself, to discover that these orthographic chunks are meaningful units.

Recent work on skilled reading in Tagalog has provided behavioural and neural evidence that acquired morphological knowledge allows skilled readers to parse infixed words (Cayado et al., 2023, 2026) and reduplicated words (Wray et al., 2022) into constituent morphemes, suggesting that morphological analysis is much more robust and flexible than previously hypothesized. Note that *<in>* and *<um>* infixes always appear after the first consonant. Skilled Tagalog readers may exploit this orthographic regularity (e.g., consonant+*in* or consonant+*um*) to trigger segmentation, and to prepare for a disrupted stem. Further, repetition of CV cluster (e.g., *tata* and *lulu* in *tatawag* and *lulundag*, respectively) in letter-strings may trigger segmentation, as it reliably signals morphological complexity. However, these studies focused on the morphological analysis of existing words, which theoretically could occur following the analysis of whole forms. The analysis of morphological structure in unfamiliar forms requires readers to have acquired abstract knowledge of morphemes that can be generalised. In this study, we investigate whether Tagalog readers possess this knowledge by testing whether they analyse morphologically structured nonwords in terms of their constituents.

Using the morpheme interference paradigm in Tagalog, we investigate whether the lack of a consistent surface realisation in infixation and reduplication negatively impacts morphological analysis during prelexical processing. We also include two highly frequent prefixes (*ka-, na-*) and suffixes (*-in, -an*) that operate in a manner similar to English prefixation and suffixation as a manipulation check in case infixes and reduplicants do not show morpheme interference effects. Following previous morpheme interference experiments, we hypothesize that adult readers should make more errors and take longer to reject morphologically structured nonwords (e.g., *woodness*) compared with their orthographic controls (e.g., *woodnels*), reflecting readers’ acquired sensitivity to morphological structure (i.e., morpheme interference effects). If stem disruption due to infixation negatively impacts morphological analysis, then morpheme interference effects should not be obtained for infixes. However, if readers are sensitive to morphological structure regardless of stem disruption, then morpheme interference effects should be obtained for infixes. Further, if morphological knowledge can be formed by tracking a consistent *pattern* onto meaning (as opposed to a consistent orthographic form), then morpheme interference effects should be obtained for reduplicants. However, if acquisition of morphological knowledge requires a mapping between a consistent orthographic form onto meaning, then no morpheme interference effects should be obtained for reduplicants.

## Methods

### Participants

Eighty participants (mean age = 31.27 years, SD = 9.76) were recruited for the experiment via Prolific. All participants were self-reported native Tagalog speakers, and reported a place of birth in which Tagalog is the dominant language. All participants also reported speaking English. Others additionally spoke another Philippine language (*N* = 25), Italian (*N* = 2), Spanish (*N* = 3), Korean (*N* = 3), and Japanese (*N* = 2). All participants reported completing their primary and secondary education in the Philippines. Three participants were excluded due to low accuracy (<60%) and replaced.

The sample size was determined by the need to maximise the number of observations per condition, while keeping the experiment short enough to prevent fatigue. We did not conduct an a priori power analysis. Instead, we followed Brysbaert and Stevens (2018) recommendation of including at least 1,600 observations per condition in a factorial design. Our experiment exceeded this, with 5,775 (morphologically structured nonword) and 5,975 (orthographic control) observations for the accuracy data once data points that clearly fell outside of the distribution were removed. For response time data, we have 4,348 (morphologically structured nonword) and 5,683 (orthographic control) observations once responses for inaccurate trials were removed. All participants gave their informed consent and they were paid £3.50. This study received a favourable opinion from the research ethics committee of Royal Holloway, University of London. 

### Materials

Morphologically structured nonwords and control nonwords without any morphological structure were created in this experiment. First, we selected eight morphological types including two prefixes (*ka-* and *na-*), two suffixes (*-in* and *-an*), two infixes (*<in>* and *<um>*), and partial and full reduplicants. We chose these morphemes as they had reasonably high type frequency based on a Tagalog corpus composed of books for elementary and high school students, suggesting that they would be familiar to participants (see Supplementary Materials for lexical statistics). These morphological types also have clear semantic and grammatical restrictions in Tagalog, which allows us to create stimuli that are unambiguously nonwords. Specifically, these morphological types mostly attach to verbal stems, hence we chose nominal and adjectival stems in this experiment. Second, each of these eight morphological types was attached to 80 distinct stems in Tagalog that are between four and eight letters long (e.g., *martes* ‘Tuesday’, *pusa* ‘cat’) to create an initial set of 640 morphologically structured nonwords. These 640 morphologically structured nonwords were then distributed across 80 participants, with each participant seeing each morphological type with 10 different stems. The affix-stem combinations in the morphologically structured nonwords differed across participants to minimise the influence of any idiosyncratic affix–stem combinations. We ensured that none of the morphologically structured nonwords exist as real words by checking each against a Tagalog corpus in SketchEngine (Kilgarriff et al., 2004).

Each participant saw 80 morphologically structured nonwords grouped into four conditions (20 each): [1] infixation, in which infixes *<in>* and *<um>* were inserted into existing stems (e.g., *m****in****artes*, *p****um****usa* from the stems *martes* ‘Tuesday’ and *pusa* ‘cat’, respectively); [2] reduplication, involving either partial reduplication (copying the initial consonant-vowel cluster (CV) of the stem, e.g., ***ma****martes*, ***pu****pusa*) or full reduplication (copying the entire stem, e.g., ***martes****martes*, ***pusa****pusa*); [3] prefixation, in which prefixes *ka-* and *na-* were attached to existing stems (e.g., ***ka****martes*, ***na****pusa*); and [4] suffixation, in which suffixes *-an* and *-in* were attached to existing stems (e.g., *martes****in***, *pusa****an***). Eighty orthographic controls were also created for each condition by modifying one letter in the affix or the reduplicant of the morphologically structured nonwords (see Crepaldi et al., 2010). Specifically, the first vowel of the affix and the reduplicant was replaced (e.g., ka- vs. ki-, <in> vs. <un>). These modified non-affixes were paired with the same monomorphemic words used for the morphologically structured nonwords. Therefore, each participant saw the same stem twice, once in a morphologically structured nonword and once in an orthographic control, with each occurrence arising in different blocks of trials. All nonwords were six to 12 letters in length and were both orthographically and phonotactically legal by following Tagalog’s syllabic and phonotactic structure.

To ensure that there was an equal number of ‘yes’ and ‘no’ responses, we included 80 monomorphemic and 80 multimorphemic existing Tagalog words. These real words were matched to nonwords in length. The multimorphemic words used the same affixes and reduplicants as the morphologically structured nonwords.

Each participant saw 320 trials in total: 160 nonwords (‘no’ responses expected) and 160 real words (‘yes’ responses expected). The nonwords included 80 morphologically structured nonwords, with each affix and reduplicant paired with 10 different stems, and 80 orthographic control nonwords. The existing words included 80 morphologically complex words and 80 monomorphemic words. All participants saw the same existing words. The stimuli were pseudorandomised for each participant to ensure that (a) no more than five words or nonwords and (b) no more than two stimuli with the same affix appeared consecutively.

### Procedure

The experiment was created and administered using Gorilla (https://www.gorilla.sc/). Each trial began with a fixation cross presented in the centre of the screen for 500 ms, followed by a lowercase word or nonword (font size 60 px). The letter string remained on the screen until a response was made. Participants were asked to press ‘A’ on the keyboard if they thought that the letter string was a real word, or the letter ‘L’ if they thought that the letter string was not a real word. Participants were instructed to respond as quickly and as accurately as possible. The next trial started 1,000 ms after participants made a response. The experiment started with six practice trials and included a break halfway through the experiment (i.e., after 160 consecutive trials). The entire experiment took 20 minutes to complete.

### Data analysis

The data were analysed in R (Version 4.2.1; R Core Team, 2022). We first examined the distribution of RTs across participants and items prior to conducting any statistical analyses. Data points that clearly fell outside of the distribution—responses faster than 200 ms or slower than 3,000 ms—were removed, resulting in the exclusion of 1,317 data points (5.14%). Data for existing words (the expected ‘yes’ responses) were excluded from any analyses. The Box-Cox test (Box & Cox, 1964) showed that an inverse transformation was appropriate for RT data. Only RTs for correct ‘no’ responses were included in the analyses, resulting in the exclusion of 1,719 data points (6.71%). Accuracy and RTs were analysed using (generalised) linear mixed-effects modelling as implemented in the *lme4* package (Bates, et al., 2015). Following Bates et al. (2018), we selected the most parsimonious model for each analysis by determining the maximal random-effects structure (for the location parameter) justified by the design and supported by the data.

The models included Condition (morphologically structured nonword vs. orthographic control nonword) and Morphological Type (Infixation, Reduplication, Prefixation, Suffixation) as fixed effects and their interaction. Condition was sum-contrast coded (0.5 for morphologically structured nonword, –0.5 for orthographic controls). We used log-likelihood ratio tests for significance testing, comparing models with and without the interaction (Condition × MorphType vs. Condition + MorphType) and with and without the fixed effect of interest using the ‘anova()’ function. Multiple comparison correction was done using the *emmeans* package in R (Lenth et al., 2022). Both accuracy and RT models included correlated varying intercepts and slopes for participants and varying intercepts for items. The final model was coded in R as InvRT ~ Condition × MorphType + (1 + Condition | Participant) + (1 | Item) for RT data, and as ACC ~ Condition × MorphType + (1 + Condition | Participant) + (1 | Item) for accuracy data. The data, scripts, and experimental materials are publicly available on the Open Science Framework page (https://osf.io/e2tjz).

## Results

**Fig. 1:**
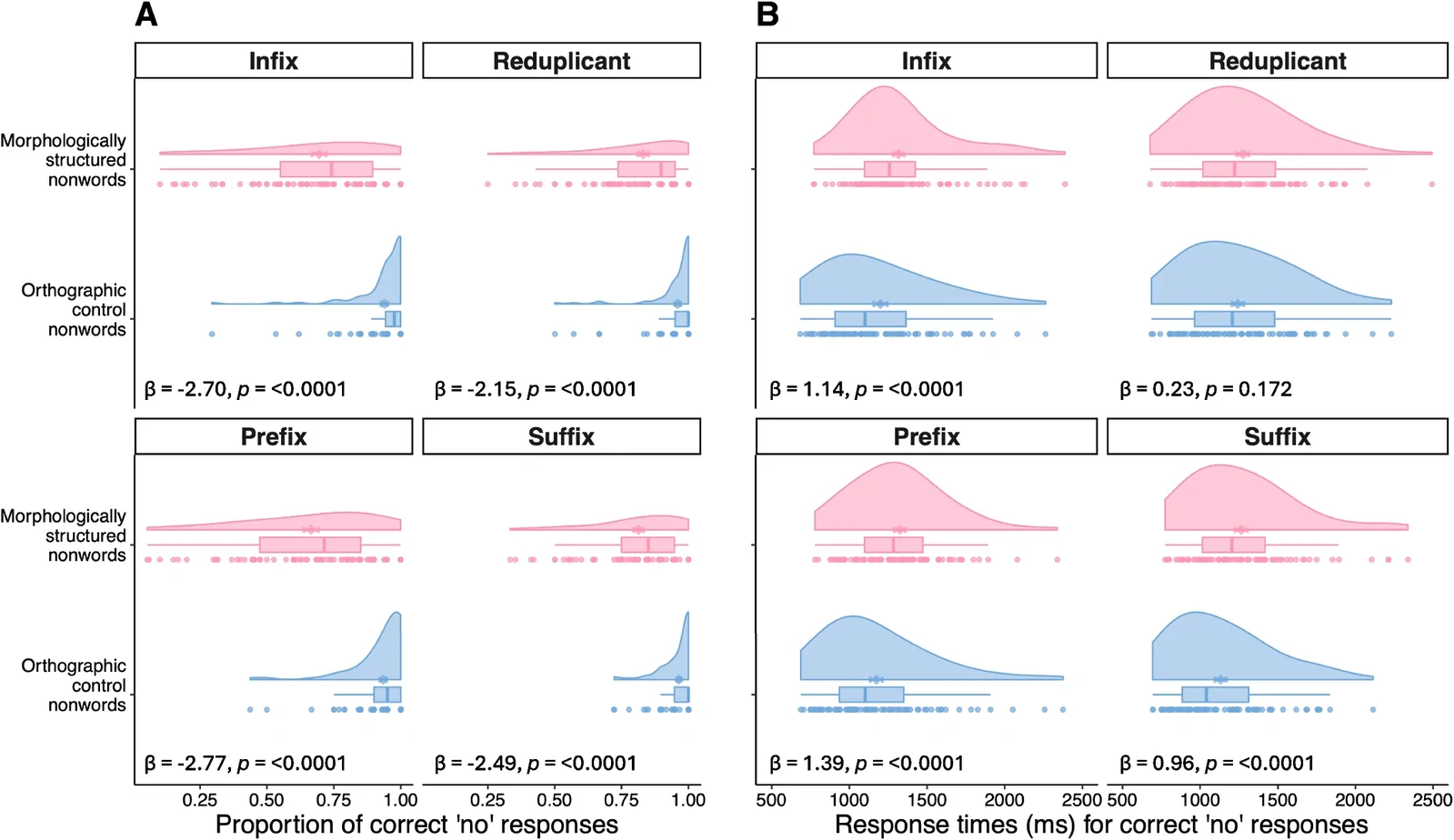
The morpheme interference effect on accuracy (**A**) and reaction times (**B**) based on mixed-effects models. On each panel, the dots represent the average for each participant. The box-and-whisker plots display the four quartiles of the ordered data, with the vertical lines inside the boxes indicating the medians (50%) and the edges of the boxes representing the lower and upper 25% quartiles. The whiskers show the expected variation in the data. The clouds illustrate the estimated data distribution, along with the means (represented by the dots) and one standard error of the mean (the error bars). Statistical analyses are reported at the bottom of each panel

Figure 1 visualises accuracy and RTs for correct ‘no’ responses to morphologically structured nonwords and their orthographic controls across four conditions. For accuracy, we found no significant interaction between Condition and MorphType, χ^2^(4) = 5.133, *p* = 0.1623, but there were significant effects of Condition, χ^2^(4) = 145.2, *p* < 0.001, and MorphType, χ^2^(4) = 106.2, *p* < 0.001. As shown in Fig. 1A, participants made *more* errors rejecting the morphologically structured nonwords than the orthographic control nonwords across four conditions: Infixation (β = −2.70, *SE* = 0.22, *p* ≤ 0.0001), Reduplication (β = −2.15, *SE* = 0.24, *p* ≤ 0.0001), Prefixation (β = −2.77, *SE* = 0.21, *p* ≤ 0.0001), Suffixation (β = −2.49, *SE* = 0.25, *p* ≤ 0.0001).

For RT, we found a significant interaction between Condition and MorphType, χ^2^(4) = 32.51, *p* < 0.001, and significant main effects of Condition, χ^2^(4) = 52.69, *p* < 0.001, and MorphType, χ^2^(4) = 43.10, *p* < 0.001. As shown in Fig. 1B, participants were *slower* to reject morphologically structured nonwords compared to nonwords with no morphological structure in Infixation (β = 1.146, *SE* = 0.17, *p* ≤ 0.0001), Prefixation (β = 1.394, *SE* = 0.17, *p* ≤ 0.0001), and Suffixation (β = 0.963, *SE* = 0.16, *p* ≤ 0.0001) conditions. No reliable morpheme interference effects were observed on RT in the Reduplication condition (β = 0.231, *SE* = 0.16, *p* = 0.172).

**Fig. 2:**
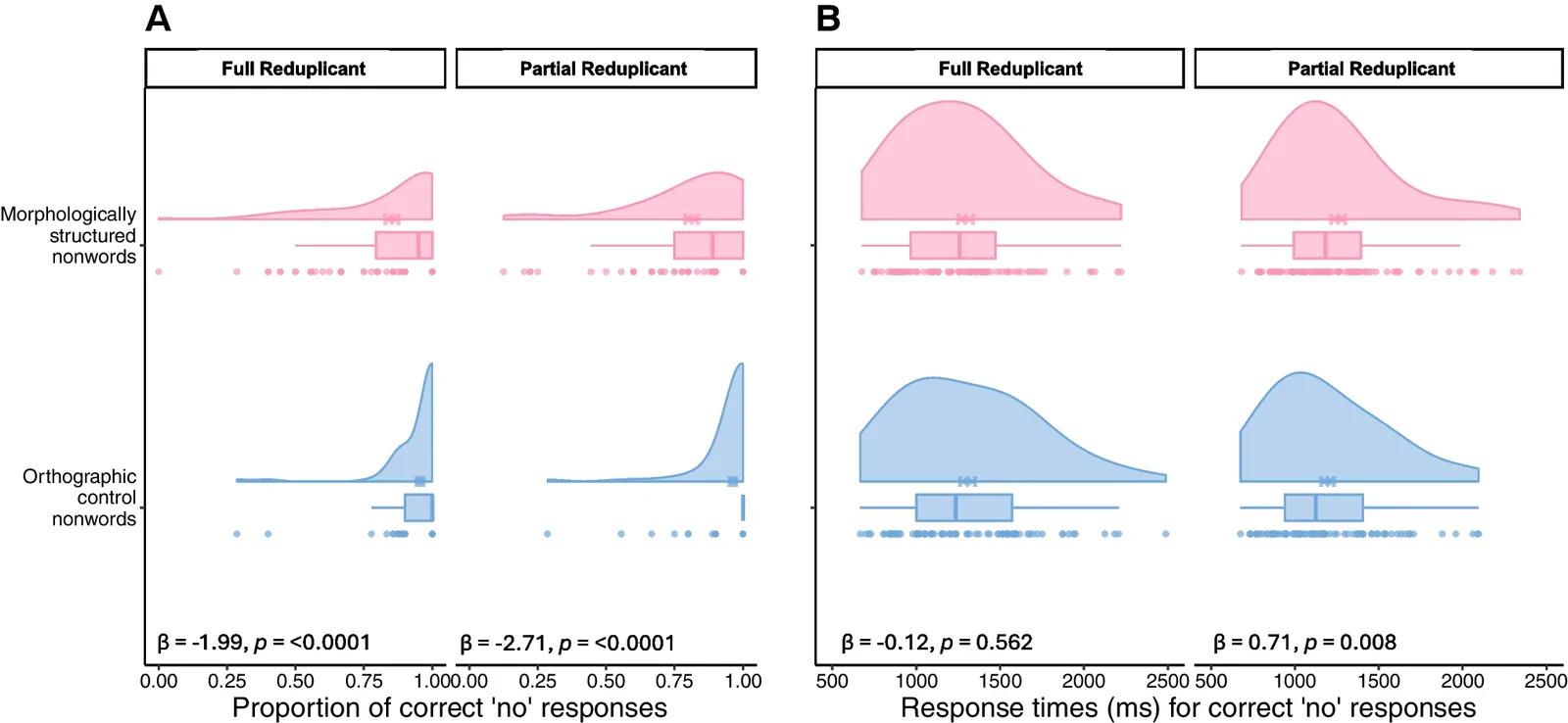
The morpheme interference effect on accuracy (**A**) and reaction times (**B**) for full and partial reduplication based on mixed-effects models. On each panel, the dots represent the average for each participant. The box-and-whisker plots display the four quartiles of the ordered data, with the vertical lines inside the boxes indicating the medians (50%) and the edges of the boxes representing the lower and upper 25% quartiles. The whiskers show the expected variation in the data. The clouds illustrate the estimated data distribution, along with the means (represented by the dots) and one standard error of the mean (the error bars). Statistical analyses are reported at the bottom of each panel

Using the same model structure as the previous analysis with Morphological Type now called Reduplicant Type, we conducted follow-up exploratory analyses in the Reduplication condition to further probe whether the morpheme interference effect is modulated by the type of reduplication (Full Reduplication vs. Partial Reduplication). For accuracy, there was no significant interaction between Condition and Reduplicant Type, χ^2^(4) = 2.77, *p* = 0.095, but there was a significant effect of Condition, χ^2^(4) = 48.64, *p* < 0.001. There was no significant effect of Reduplicant Type, χ^2^(4) = 0.52, *p* = 0.470. As shown in Fig. 2A, participants committed *more* errors rejecting morphologically structured nonwords compared to orthographic controls in both conditions (Full Reduplicant: β = −1.99, *SE* = 0.35, *p* ≤ 0.0001; Partial Reduplicant: β = −2.71, *SE* = 0.36, *p* ≤ 0.0001). For RT, we found a significant interaction between Condition and Reduplicant Type, χ^2^(4) = 8.637, *p* = 0.003, while the main effects of Condition, χ^2^(4) = 3.397, *p* = 0.065, and Reduplicant Type, χ^2^(4) = 3.582, *p* = 0.058, did not reach the significance criterion. Figure 2B shows that participants were slower rejecting morphologically structured nonwords than orthographic controls in Partial Reduplicant (β = 0.717, *SE* = 0.21, *p* = 0.0008), while no such effects were found in Full Reduplicant (β = −0.126, *SE* = 0.21, *p* = 0.562).

## Discussion

There is a large body of research suggesting that readers engage in prelexical morphological processing, but this evidence base consists of a narrow range of morphological types. We were interested in Tagalog infixation and reduplication because these morphological processes undermine readers’ ability to track stable orthographic forms with similar meanings or meaningful functions—a key requirement of statistical learning theories of morphology (Korochkina & Rastle, 2025; Plaut & Gonnerman, 2000). Results showed robust morpheme interference (MI) effects: participants made more errors and took longer to reject nonwords with morphological structure compared to those without, indicating that adult readers in our sample had acquired sensitivity to the four different morphological types tested and could apply this abstract knowledge to unfamiliar forms. MI effects found in prefixation and suffixation are consistent with results from linear morphologies in English and related European languages (e.g., Carden et al., 2019; Crepaldi et al., 2010; Dawson et al., 2018; Korochkina et al., 2026). More importantly, MI effects in infixation and reduplication substantially extend the typological scope of the morpheme interference paradigm and prompts necessary revisions to existing accounts of morphological analysis and learning.

Existing theoretical accounts propose that readers discover orthographic sequences that contribute predictably to the meanings of words (Korochkina & Rastle, 2025; Plaut & Gonnerman, 2000). Our findings from infixation inform these theories: we observed robust morpheme interference effects for infixed words in both accuracy and response time. Morpheme interference effects have also been observed in Hebrew (Yablonski & Ben-Shachar, 2016), where roots consist of non-contiguous consonant strings (e.g., *g-d-l* root in *gadol* ‘big’ and *gadal* ‘he grew’). However, the case of infixation in Tagalog is different since infixes transform typically contiguous stems into non-contiguous ones. This result indicates that adult readers successfully detect morphological structure even when the stem is disrupted; for example, a stem appearing as *t*–*awag* (i.e., disrupted form) in *tinawag* ‘called’ instead of *tawag* in *nagtawag* ‘called for’. These results are consistent with previous evidence from Tagalog infixation using existing morphologically complex words (Cayado et al., 2023, 2026; Wray et al., 2022). Note that the way stems are disrupted due to infixing follows a predictable and frequent pattern—infixes *-in-* and *-um-* are always inserted after the first consonant. Such orthographic sequence potentially signals that a broken form of the stem is coming. It may be that frequent encounters with this orthographic sequence (i.e., consonant+*in* or consonant+*um*) results in the development of representations of letter position that are sufficiently flexible for readers to detect affixes in a less salient position (i.e., not at the edge) and recognise that the stem in *na-tawag* ‘called (accidentally)’ is the same as in *t-in-awag* ‘called’ (i.e., *tawag*). Further, this orthographic sequence also typically only appears in infixation, making it a reliable cue to complexity that skilled Tagalog readers can exploit to detect both the infix and the disrupted stem.

Our findings regarding reduplication also inform statistical learning accounts because this is a case in which there is no consistent surface realisation of the morpheme at all. Our findings imply that readers need not track stable orthographic chunks; instead, they can exploit consistent orthographic *patterns* that predictably contribute to the meaning of the word. In Tagalog, readers appear to track the systematic copying of CV sequences in ***lu****lundag* ‘will jump’ ***ta****tawag* ‘will call’ and ***ti****tili* ‘will scream’ that signal ‘incomplete action’, allowing them to build a representation of reduplicants as an abstract CV skeleton in the mental lexicon. Processing partially reduplicated words requires readers to slot the reduplicants *lu-*, *ta-*, and *ti-* onto this abstract CV skeleton (Marantz, 1982). Further, skilled Tagalog readers may initiate morphological analysis as soon as a CV sequence is repeated in a letter-string, treating them as strong cues to morphological complexity. This pattern tracking is even more complicated in words consisting of both partial reduplication and infixation (e.g., *t<in>a-tawag* ‘was being called’). Perhaps these cases may first require detecting the infix <in> after the first consonant, thereby leaving the string *tatawag*, which will then allow the tracking of CV reduplicant. Further experiments are required to examine how these more complex words are processed by readers. Together, our results demonstrate that sensitivity to morphological structure is not contingent on stable orthographic forms.

How, then, do readers of Tagalog learn to recognise these morphemes? Korochkina and Rastle (2025) proposed that the ease with which morpheme representations are acquired depends on the relationship between the number of exemplars with genuine morphological structure (e.g., *teacher* and *singer* both use -*er* to communicate agentive status) and the number of morphological false alarms (e.g., *brother* is a morphological false alarm as it has *broth-* and *-er* despite being monomorphemic). Morphemes that occur frequently in transparent morphological contexts and that participate in few false alarms show more robust representations (Korochkina et al., 2026). Infixes and reduplicants in Tagalog are also highly frequent and productive with very few morphological false alarms, and they carry important grammatical information in the language. The combination of these factors means that children learning Tagalog will have robust knowledge of these morphemes even before they learn to read (see Garcia & Kidd, 2020, for production evidence of acquisition of infixes in young children at the sentence level). Consequently, readers of Tagalog may acquire knowledge of infixes and reduplicants relatively easily because they reliably contribute to word meaning, with very little noise in the input, and they carry robust morphological knowledge they acquired from spoken language. The combination of these factors may facilitate efficient morphological learning and analysis, mitigating the potential impact of disrupted and unstable orthographic forms.

In conclusion, our study represents a critical step toward a more general understanding of how morphemes are processed and acquired. Examining understudied languages such as Tagalog, alongside underexamined morphological types like infixation and reduplication, allows us to test the boundary conditions of claims around morphological learning and processing. By moving beyond suffixation, the present findings not only test the limits of current accounts but also raise a new question about whether morphological knowledge can be acquired by tracking consistent *patterns*. We show that having stable orthographic forms is not a key requirement when building morphological knowledge. Instead, morphological analysis and learning appear more robust and flexible than previously assumed, such that infixes and partial reduplicants are successfully detected and learned, and skilled Tagalog readers are able to apply abstract knowledge of these morphemes to analyse unfamiliar words. However, morphological analysis appears less robust for full reduplicants, likely due to their lower type frequency relative to partial reduplicants (see Supplementary Materials for type frequency of different morphological types). Future research should therefore examine how type frequency influences morpheme detectability, while also leveraging other understudied morphological types to test the generalisability of existing claims and to generate new questions about morphological learning and processing.

## Supplementary Information

Below is the link to the electronic supplementary material.

Supplementary file1 (DOCX 15 KB)

## Data Availability

The data and experimental materials are available on OSF (https://osf.io/e2tjz).

## Availability of code

All analysis codes are available on OSF (https://osf.io/e2tjz).
