# Supplementary material for: Morphological analysis when morphemes are hard to detect: Evidence from infixation and reduplication
Source: Psychon Bull Rev. 2026 Oct 23;33(8):262. doi: 10.3758/s13423-026-03026-y (PMC13694315; doi:10.3758/s13423-026-03026-y)
Supplement: Supplementary file 1 [file 13423_2026_3026_MOESM1_ESM.docx]

Supplementary materials showing type and per million frequencies of each morphological type

| **Morphological Type** | **Type Frequency** | **Per Million Frequency** |
| --- | --- | --- |
| ka- Prefix | 3262 | 2092.3 |
| na- Prefix | 4449 | 2853.6 |
| <in> Infix | 3598 | 2307.7 |
| <um> Infix | 2882 | 1848.6 |
| -an suffix | 2008 | 1287.9 |
| -in suffix | 455 | 291.9 |
| Partial reduplication | 7618 | 4886.1 |
| Full reduplication | 1094 | 701.7 |
